# Demethylation of Wheat Straw Alkali Lignin for Application in Phenol Formaldehyde Adhesives

**DOI:** 10.3390/polym8060209

**Published:** 2016-05-30

**Authors:** Yan Song, Zhixin Wang, Ning Yan, Rong Zhang, Jinchun Li

**Affiliations:** 1 Faculty of Materials Science & Engineering, Changzhou University, Changzhou 213164, China; ysong@cczu.edu.cn (Y.S.); wzx911003@163.com (Z.W.); rzhang@cczu.edu.cn (R.Z.); 2 Jiangsu Collaborative Innovation Center of Photovolatic Science and Engineering, Changzhou University, Changzhou 213164, China; 3 Faculty of Forestry, University of Toronto, 33 Willcocks Street, Toronto, ON M5S 3B3, Canada; ning.yan@utoronto.ca

**Keywords:** wheat straw alkali lignin, Lewis acid, demethylation, phenol formaldehyde adhesive

## Abstract

Lignin is a natural biopolymer with a complex three-dimensional network. It is the second most abundant natural polymer on earth. Commercially, lignin is largely obtained from the waste liquors of pulping and bioethanol productions. In this study, wheat straw alkali lignin (WSAL) was demethylated by using an *in-situ* generated Lewis acid under an optimized demethylation process. The demethylation process was monitored by a semi-quantitative Fourier Transform Infrared Spectroscopy (FTIR) method. The demethylated wheat straw alkali lignin (D-WSAL) was further characterized by Proton Nuclear Magnetic Resonance (^1^H NMR), Gel Permeation Chromatography (GPC), and titration methods. After the demethylation process, it was found that the relative value of the methoxy group decreased significantly from 0.82 to 0.17 and the phenolic hydroxyl group increased from 5.2% to 16.0%. Meanwhile, the hydroxyl content increased from 6.6% to 10.3%. GPC results suggested that the weighted averaged molecular weight of D-WSAL was lower than that of WSAL with a smaller polydispersity index. The D-WSAL was then used to replace 60 wt % of phenol to prepare lignin-based phenol formaldehyde adhesives (D-LPF). It was found that both the free formaldehyde content and the free phenol content in D-LPF were less than those of the lignin-based phenol formaldehyde adhesives without lignin demethylation (LPF). Gel time of D-LPF was shortened. Furthermore, the wet and dry bonding strengths of lap shear wood samples bonded using D-LPF were higher than those of the samples bonded using LPF. Therefore, D-WSAL has shown good potential for application in phenol formaldehyde adhesives.

## 1. Introduction

Adhesives are key components in the manufacturing of particleboards, wood panels, fiberboards, and plywood. Among various adhesives, the resole phenol formaldehyde (PF) is most commonly used for exterior applications [1,2]. Commercial PF adhesives are synthesized mostly using petroleum-derived feedstock. With the dwindling petroleum resources [3,4], there is a growing interest to explore alternative raw materials from renewable resources to make bio-based adhesives [5,6,7,8].

**Figure 1:**
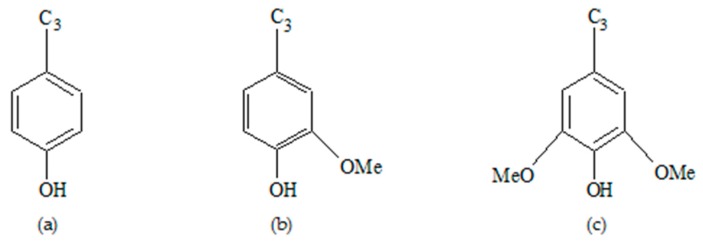
Three kinds of phenyl propane monomer units of lignin, including (**a**) p-hydroxyphenyl propane (H); (**b**) guaiacyl propane (G), and (**c**) syringyl propane (S).

Lignin is a natural polymer with a three-dimensional network structure that consists of p-hydroxyphenyl propane, guaiacyl propane, and syringyl propane units (Figure 1). The three units are connected through C–C and C–O–C [9,10] bonds. Furthermore, lignin is one of the most abundant organic polymeric materials on earth, only second to cellulose. Commercial lignin is mainly obtained from waste liquor in the pulping industry or as a by-product of bio-ethanol production [11,12].

It has been estimated that the lignin disposal as waste from the global forest industry has increased to 2000 million tons annually in recent years, with wheat straw alkali lignin accounting for 32% of the total amount. Only less than 2% of the lignin is reused as organic materials [13]. Most lignin from the forest industry is either burned or directly disposed of in the form of liquid waste [14]. Lignin consists of functional groups exhibiting similar properties to phenol, therefore it is considered the most promising substitute for phenol targeting adhesive applications [15,16]. However, lignin is a large molecular weight polymer with a complex structure [17] which has low reactivity towards formaldehyde. In order to enhance the reactivity, many efforts were made to modify lignin, for example, by methylolation, demethylation, and phenolation [16,18,19,20]. Among these modifications, demethylation has been shown to be an effective method to increase the reactivity of lignin. It has been reported that demethylation of Kraft lignin using sulfur at 225–235 °C under high pressure enhanced lignin reactivity in phenol formaldehyde adhesives [21]. Later, Wu and Zhang [22] demethylated wheat straw soda lignin using a similar method for preparing lignin-based phenolic formaldehyde adhesives.

Meanwhile, Lewis acids have been successfully applied in demethylation of methoxyl groups in small molecules. Lignin has many methoxyl groups and it has been shown that lignin can also be demethylated using a Lewis acid [23,24]. Furthermore, Chung and Washburn [25] reported demethylation of softwood kraft lignin (SKL) by hydrobromic acid and phase transfer catalyst hexadecyltri-*n*-butylphosphonium bromide (TBHDPB) in a H_2_O–DMF mixture. The hydroxyl group in SKL increased by 28% and the demethylated SKL was more reactive than the unmodified SKL when used for polyurethane preparation. Moreover, Ferhan *et al.* [26] investigated the demethylation of SKL by using *in-situ* generated hydro-iodic acid, and concluded that the total hydroxyl number of D-SKL increased by 60% as measured by ^31^P NMR. So far, there have been few reports on the demethylation of wheat straw alkali lignin (WSAL) by using Lewis acid generated *in-situ*. WSAL has a higher molecular weight and a higher content of syringl propane units compared with SKL. One important consideration is that WSAL constitutes the majority share of the lignin productions in China. It is valuable to investigate demethylation of WSAL for application in phenol formaldehyde adhesives [27,28].

In this study, WSAL was demethylated by using iodocyclohexane (ICH), and the modification efficacy was evaluated with a Fourier Transform Infrared Spectroscopy (FTIR) semi-quantitative method. The demethylated WSAL was characterized by FTIR, ^1^H NMR, and Gel Permeation Chromatography (GPC) respectively. The hydroxyl group content was quantified by a titration method. Finally, the demethylated WSAL was used to prepare lignin-based phenol formaldehyde adhesives (D-LPF).

## 2. Materials and Methods

### 2.1. Materials

Wheat straw alkali lignin (WSAL) (hydroxyl group content: 6.65 wt %, *M*_W_: 6000 g/mol, polydispersity index: 1.3, density: 1.30 g/cm^3^) was purchased from Shandong Quanlin Paper Co., Ltd., Gaotang, China. *N*,*N*-dimethyl formamide, dichloromethane and ethyl ether (AR grade) were purchased from Strong Functional Chemical Co., Ltd., Changshu, China. Iodocyclohexane was purchased from Tokyo Chemical Plant Type Club (Tokyo, Japan). Formaldehyde solution (37 wt %), phenol and sodium hydroxide (NaOH) were of analytical grade and purchased from Medicine Group Chemical Reagent Co., Ltd., Shanghai, China. All chemicals were used without further purification.

### 2.2. Demethylation of WSAL

**Scheme 1:**
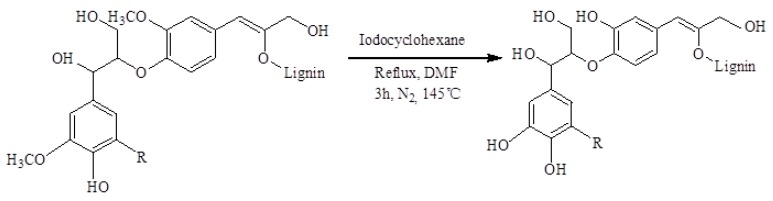
Demethylation route of WSAL (**R** represents H, OH or –OCH_3_). (demethylation was carried out with 10; 1 molar ratio of ICH to lignin at 145 °C for 3 h with 4 mL DMF under nitrogen atmosphere).

Demethylation of WSAL was carried out as follows (Scheme 1): WSAL, *N*,*N*-dimethyl formamide (DMF) and iodocyclohexane were charged into a 50 mL three-neck round bottom flask equipped with a reflux condenser, a magnetic stirrer, and a thermometer. The mixture was stirred and heated to 145 °C, and was kept under a nitrogen atmosphere for 3 h. The mixture was then cooled down to room temperature to terminate the reaction and diluted with a proper amount of dichloromethane. Subsequently, the solvent in the mixture was evaporated under vacuum to obtain the crude product, which was washed twice with 100 mL deionized water each time. The heterogeneous mixture in water was centrifuged at 5000 rpm. The precipitate was dried to a constant weight in a vacuum oven at 80 °C and stored in a desiccator for further characterization.

### 2.3. Preparation of Adhesives

Phenol formaldehyde (PF) and lignin phenol formaldehyde(LPF) adhesives were prepared in a 100 mL four-neck round bottom flask equipped with a stirrer, a reflux condenser and a thermometer.

#### 2.3.1. Preparation of PF Adhesive

PF adhesive was prepared according to [29]. Firstly, 0.22 g NaOH, 1.20 g phenol, and 0.90 g distilled water were added into a flask, stirred and heated up to 50 °C for 10 min. Subsequently, 2.03 g formaldehyde was added dropwise in 5 min. Finally, the mixture was then heated at 90 °C for 3.5 h.

#### 2.3.2. Preparation of LPF or D-LPF Adhesive

LPF adhesives were prepared in two steps: the first step was similar to the preparation of PF adhesives as described above, in which 40% of phenol was added, and the reaction continued for 1.5 h; Secondly, D-WSAL or WSAL with the substitution of 60 wt % of phenol, was added to the mixture, and the reaction was kept for 2 h.

### 2.4. Preparation of Plywood

**Figure 2:**
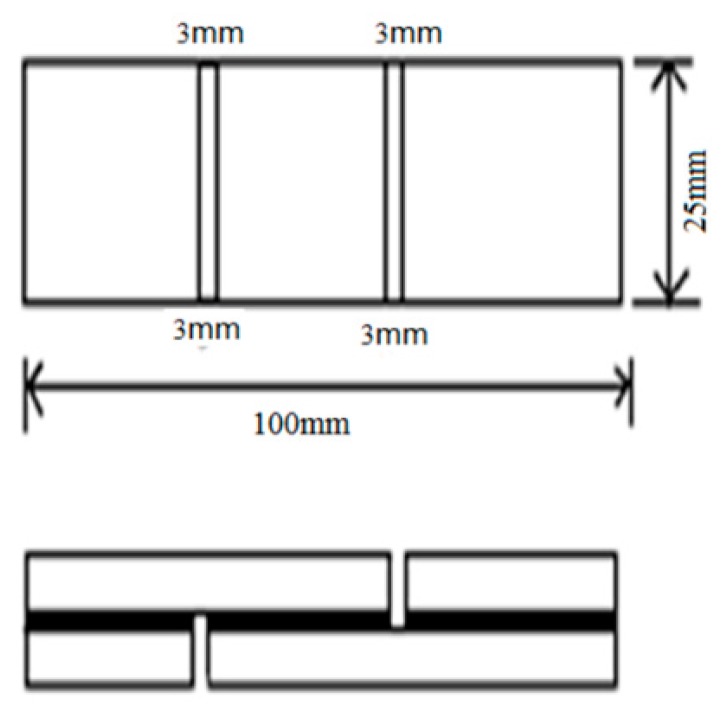
Sample schematic for lap-shear testing (100 mm × 25 mm × 4.0 mm), with two layers of pine veneers coated with one layer of adhesive.

The PF, D-LPF, and LPF adhesives synthesized above were used to prepare lap shear specimens for bonding tests according to the procedure specified by the Chinese National Standard (GB/T 14732-2006 type I plywood). Standard single-layer pine veneers (100 mm × 25 mm × 4.0 mm) were dried to 5 wt % moisture content and coated with 125 g/m^2^ adhesive to obtain the two-layer bonded specimens. Specimens with two layers of pine veneers coated with one layer of adhesive were pressed at 150 °C under a pressure of 1.0 MPa for 6 min. The bonded wood samples were kept indoors for 24 h before the lap-shear measurement (Figure 2).

### 2.5. Characterization

In this study, acetylation of D-WSAL and WSAL was carried out according to the published procedure [30] to improve the accuracy of the FTIR analysis and the degree of dissolution of the samples in solvents for ^1^H NMR and GPC analysis.

#### 2.5.1. Fourier Transform Infrared Spectroscopy (FTIR)

The Avatar370 type Fourier infrared spectrometer (Nicolet Co., Ltd., Madison, WI, USA) was used for the experiments. The testing samples were prepared by grinding a mixture of 2 mg WSAL or D-WSAL and 200 mg KBr before pressing at 10 MPa for 3 min. The scanning range was 4000–400 cm^−1^ and the resolution ratio was 4 cm^−1^.

#### 2.5.2. Nuclear Magnetic Resonance Spectroscopy (NMR)

The NMR spectra were recorded on a DRX-400 spectrometer (Bruker Co., Ltd., Fällanden, Switzerland) using DMSO-d_6_ as the solvent, and the acetylated samples were characterized by 400 M AVANCE type NMR (Bruker Co., Ltd.)

#### 2.5.3. Average Molecular Weight Determination by Gel Permeation Chromatography (GPC)

The average molecular weight of acetylated D-WSAL and its distribution were determined with a Waters 1515 gel permeation chromatography (GPC, Waters, Milford, MA, USA) and the device was equipped with a refractive index detector using HR1, HR3, and HR4 columns with molecular weights in the range of 100–500,000 g/mol, calibrated with polystyrene (PS) standard samples.

#### 2.5.4. Quantification of Hydroxyl Groups

The total hydroxyl groups of WSAL and D-WSAL were quantified by using a standard phthalation procedure as reported previously [31]. The principle is based on the reaction between the phthalation reagent and the lignin sample to create formic acid which was back-titrated with 1 mol/L NaOH standard solution to the equivalence point. The hydroxyl value was calculated according to the following Equation (1):

(1) $$\text{Hydroxyl Value }(\text{mmol}/g)\;=\;\frac{\left(V_{A}\;-\;V_{B}\right)\;\times \;N\;}{W}$$

where *V*_A_ is the volume of NaOH standard solution consumed to titrate the blank solution, mL; *V*_B_ is the volume of NaOH solution used to titrate the sample solution, mL; *N* is the normality of the NaOH solution, mol/L; and *W* is the weight of the sample, g.

#### 2.5.5. Properties of the Adhesives

The adhesive’s properties, including solids content, free phenol content, and free formaldehyde content, were determined according to the Chinese National Standards (GB/T 14074-2006). The solids content was measured by weighing 1 g of sample prior to drying to a constant weight at 120 °C, and then the percentage ratio of the weight of the dried sample to that of the initial sample was namely the solids content. The free phenol in the adhesive was distilled out by steam distillation, and the free phenol content was determined by means of titration with a saturated bromine aqueous solution. The free formaldehyde content was tested using hydroxylamine hydrochloride, and then the free formaldehyde content was titrated with sodium hydroxide solution.

The gel time was measured by charging 1 g of adhesive into a 12 mm-diameter test tube, and heating the test tube at 150 °C in an oil bath. Gel time was defined as the time period from placing the test tube into the oil bath to the initial gelation of adhesive.

#### 2.5.6. Bonding Strength of Lap-Shear Specimens

Both dry bonding strength and wet bonding strength of the lap-shear samples were tested according to the procedures specified by the Chinese National Standards (GB/T 14074-2006). An electronic universal testing machine (Shanghai Yi Huan Instrument Technology Co., Ltd., Shanghai, China) and a plate curing instrument (Shanghai Machinery Factory Co., Ltd., Shanghai, China) were used in these experiments. All the bonded lap-shear samples were handled in four steps before testing the wet bonding strength as follows: (1) Immersing in boiling water for 4 h; (2) drying at 63 °C for 20 h; (3) immersing in boiling water for 4 h; (4) cooling for 10 min. The tensile strength of the specimens was measured with an electronic universal testing machine at a constant loading speed of 10 MPa/min. The maximum load was recorded to 10 N accuracy. Two samples of each lap-shear specimens were tested for wet bonding strength.

## 3. Results and Discussion

### 3.1. Optimization of the Demethylation Process

FTIR is an effective tool to characterize the specific structural groups of polymer materials. Furthermore, the FTIR semi-quantitative method has been used to characterize the relative content of specific groups, in which the phenyl group’s skeleton vibration peak at 1503 cm^−1^ was selected as the reference for calculating the relative values of other groups by area ratios of the peaks [32]. In order to optimize the demethylation process, the influence of various factors on the demethylation reaction was investigated by using the FTIR semi-quantitative method. The factors reported here include ICH dosage based on lignin amount, reaction time, reaction temperature, and solvent types.

#### 3.1.1. Iodocyclohexane Dosage

**Figure 3:**
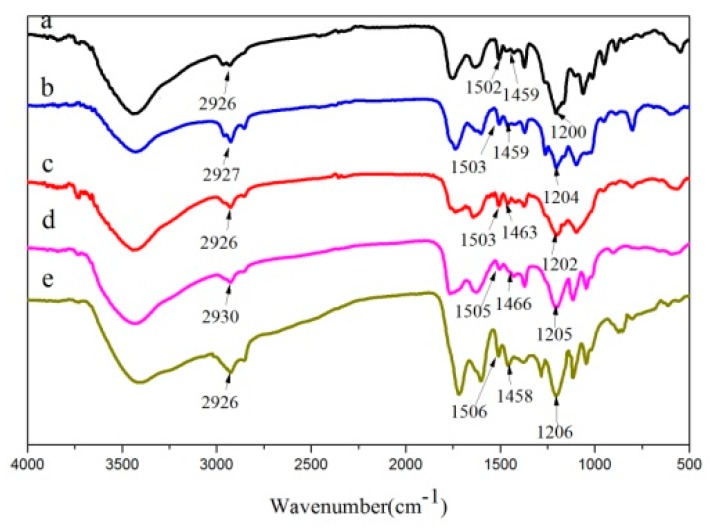
FTIR spectra of acetylated WSAL and D-WSAL with 0 (**a**), 5:1 (**b**), 10:1 (**c**), 12:1 (**d**) and 15:1 (**e**) molar ratio of ICH to lignin, prepared at 145 °C for 3 h with 4 mL DMF under nitrogen atmosphere.

Five levels of iodocyclohexane (ICH) to lignin molar ratios ranged from 5:1 to 15:1 (mol/mol) were used in this set of experiments while keeping the other factors fixed according to literature values and past experiences. Temperature was set at 145 °C and reaction time was fixed at 3 h while the volume of the solvents was 4 mL. The synthesized D-WSAL and WSAL were characterized by FTIR and analyzed with the semi-quantitative method (Figure 3, Table 1). The results showed that with an increase in the molar ratio of ICH *versus* lignin, the relative values of the methoxyl group at 1459 cm^−1^ exhibited a decreasing trend from 0.82 to 0.17 at a molar ratio of 12:1, and then leveled off. Meanwhile, the relative values of phenol hydroxyl at 1200 cm^−1^ increased from 1.01 to 11.9 at a molar ratio of 12:1, and then decreased at a higher molar ratio. It seems that overdosed ICH inhibited the demethylation reaction. Furthermore, when the molar ratio was less than 10:1, it resulted in a mixture of mono-demethylated and bis-demethylated products, inconsistent with previous reports [23]. Therefore, the molar ratio of 12:1 was chosen for the subsequent demethylation of WSAL.

#### 3.1.2. Effect of Solvent Amount

**Figure 4:**
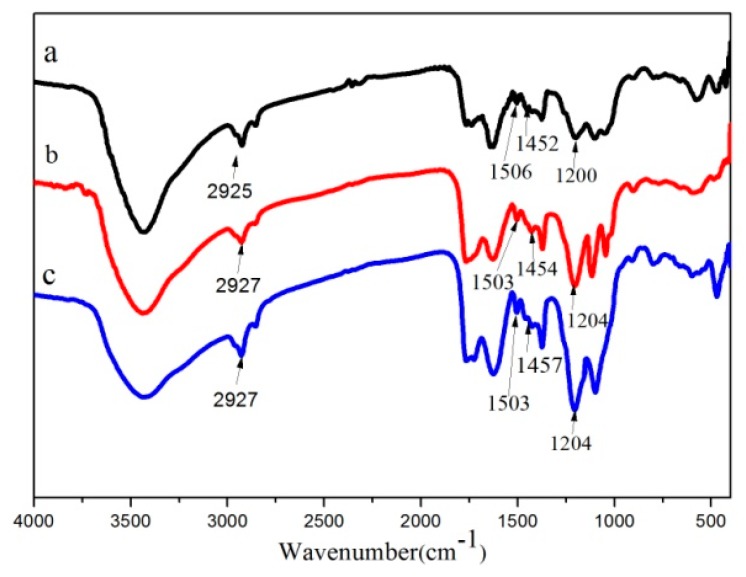
FTIR spectra of D-WSAL prepared with 2.5 mL (**a**), 4 mL (**b**), and 5.5 mL (**c**) DMF with 12:1 molar ratio of ICH to lignin, at 145 °C for 3 h under nitrogen atmosphere.

In order to investigate the effect of solvent amount on the demethylation reaction, three DMF to lignin mass ratios ranging from 11.9:1 to 26.1:1 (*w*/*w*) were used respectively in this set of experiments with an iodocyclohexane to lignin molar ratio at 12:1 (mol/mol) and the other reaction conditions kept the same as above. The synthesized D-WSALs were characterized and analyzed (Figure 4 and Table 2). The relative values of methoxyl group at 1459 cm^−1^ reached a minimum of 0.17 when the solvent dosage was 4 mL, while at the same time the relative value of phenol hydroxyl on D-WSAL at 1200 cm^−1^ reached a maximum 11.90. Therefore, it can be inferred that the amount of solvent is important for the demethylation reaction. A proper amount of solvent would result in effective reactions based on optimum contacts or collisions among different reagents [33]. The results indicated that a mass ratio of 19.0:1 (*w*/*w*) was the optimum level to be used, which was in agreement with past work [26].

#### 3.1.3. Effect of Reaction Temperature

**Figure 5:**
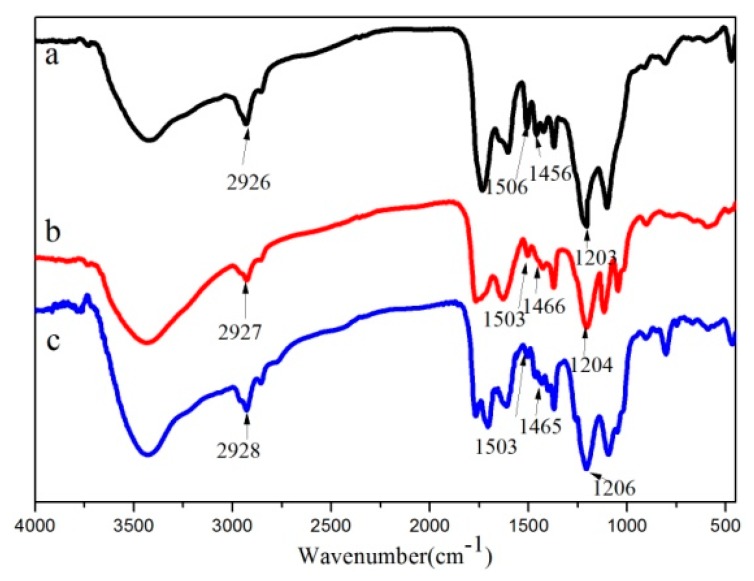
FTIR spectra of D-WSAL synthesized at 135 °C (**a**), 145 °C (**b**), and 155 °C (**c**) with 12:1 molar ratio of ICH to lignin for 3 h with 4 mL of DMF under nitrogen atmosphere.

Three reaction temperatures, specifically 130, 145 and 155 °C, were examined for the demethylation of WSAL. The solvent volume was 4 mL, the ICH/lignin molar ratio was 12/1 and other factors were kept the same. The prepared D-WSALs were analyzed the same as in 3.2.2 (Figure 5, Table 3). The lowest relative value of the methoxyl group at 1459 cm^−1^ was 0.17 at 145 °C, corresponding to the highest relative value (11.9) of phenol hydroxyl group at 1200 cm^−1^. This could be due to the cross-linking reaction among the hydroxyl groups at 155 °C, leading to a lower relative value (7.63) of phenol hydroxyl groups. However, at 135 °C, the system could not obtain enough thermal energy to overcome the reaction activation energy of the methoxy groups at various positions on WSAL to maximize the demethylation process [34]. Based on these results, 145 °C was selected as the optimum temperature for the demethylation reaction in this study.

#### 3.1.4. Effect of Reaction Time

**Figure 6:**
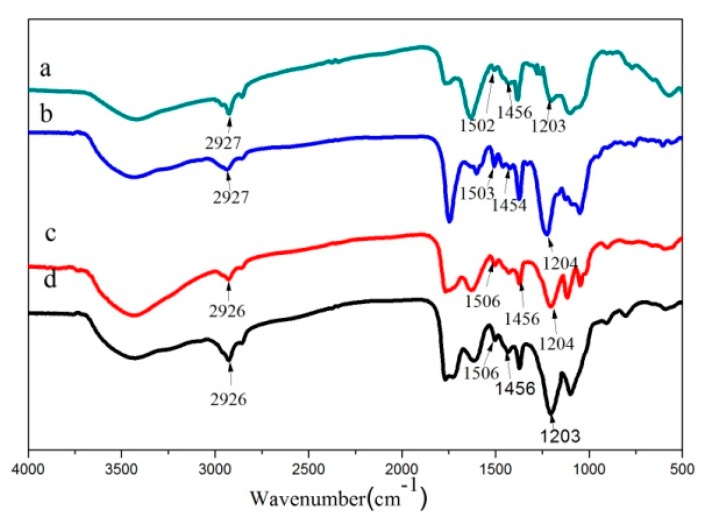
FTIR spectra of WSAL prepared for 7 h (**a**), 5 h (**b**), 3 h (**c**), and 2 h (**d**) with 12:1 molar ratio of ICH to lignin with 4 mL DMF at 145 °C under nitrogen atmosphere.

In order to investigate the effect of reaction time on the demethylation of WSAL, the reaction was carried out for 2–7 h at the reaction temperature 145 °C with the other reaction factors kept constant. The synthesized D-WSALs were analyzed and results are shown in (Figure 6, Table 4). The relative values of the methoxyl group at 1459 cm^−1^ changed little up to 7 h reaction time and the same was true for the phenol hydroxyl groups at 1204 cm^−1^. However, the maximum relative value of phenol hydroxyl group was 11.90 which was reached after 3 h of reaction. Therefore 3 h was chosen as the optimum demethylation time for WSAL. Too long a reaction time could lead to more side reactions, such as cross-linking among the hydroxyl groups [26].

### 3.2. Characterization of Demethylated Lignin

D-WSAL prepared under the optimum demethylation condition, (reaction time of 3 h, reaction temperature at 145 °C, molar ratio of ICH to lignin at 12:1 and solvent dosage of 4 mL), was characterized by FTIR, ^1^H NMR, and GPC, respectively.

**Figure 7:**
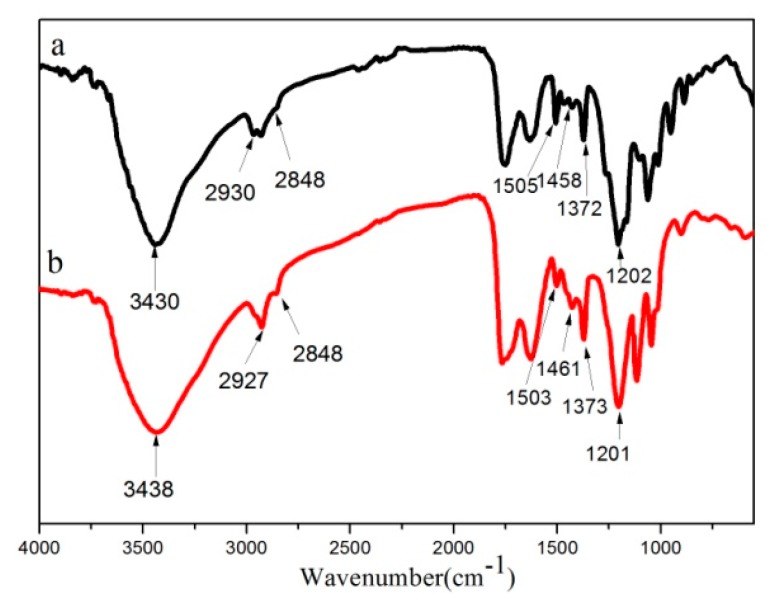
FTIR spectra of WSAL (**a**) and D-WSAL (**b**) prepared under the optimum demethylation condition: 12:1 molar ratio of ICH to lignin, 145 °C, 4 mL DMF, 3 h reaction time under nitrogen atmosphere.

The characteristic peaks of WSAL and D-WSAL obtained from FTIR spectra are shown in Figure 7, and the characteristic peak assignments are listed in Table 5. The absorption peaks at 1372, 1264 and 843 cm^−1^ showed WSAL as a typical herbaceous lignin [35]. The main difference between D-WSAL and WSAL was the peak area of phenolic hydroxyl groups (higher for D-WSAL) and methoxyl groups (reduced for D-WSAL), which is consistent with previous demethylation studies [36,37]. The FTIR semi-quantitative method was used to characterize the relative content of specific groups. The content of phenolic hydroxyl groups in D-WSAL increased from 1.01 to 11.90 for WSAL, while the methoxyl groups decreased from 0.82 to 0.17 for WSAL. The relative value of phenolic hydroxyl groups was increased by 10.89, which was higher than that of the methoxy group which decreased by 0.65. This may be due to part cleavage of ether bonds during the demethylation process [18]. The results showed that the WSAL was demethylated successfully and more efficiently than in other reported methods [21,25]. The methods reported in the past, employed demethylation processes with harsher conditions and had a lower yield of phenolic hydroxyl groups.

**Figure 8:**
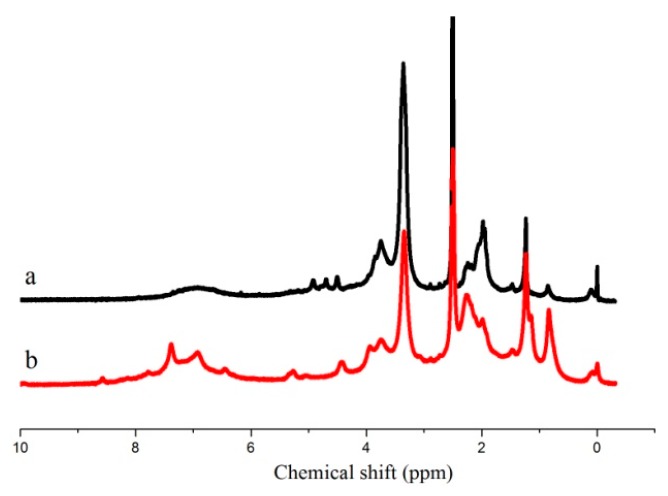
^1^H NMR spectra of WSAL (**a**) and D-WSAL (**b**) prepared under the optimum demethylation condition: the demethylation process was carried out with 12 molar ratio of ICH to lignin, prepared at 145 °C with 4 mL DMF for 3 h under nitrogen atmosphere.

D-WSAL was further characterized by ^1^H NMR. D-WSAL and WSAL were acetylated before the measurement to enhance the solubility of the samples in deuterium reagent. From the ^1^H NMR spectrum, the phenolic hydroxyl group and aliphatic hydroxyl group could be identified (Figure 8, Table 6). The chemical shift 7.80–7.34 ppm was assigned to the aromatic proton of p-hydroxyphenyl (H), and 7.34–6.80 ppm to the aromatic proton of guaiacyl propane (G), as well as 6.80–6.32 ppm to the aromatic proton of syringyl propane (S), which confirmed the herbaceous lignin’s structure of WSAL. These results are consistent with the reported result [38]. Furthermore, the percentage of hydrocarbon increased from 9.5 to 23.6, indicating that a large amount of hydrocarbons had been generated from the degradation of lignin. The percentage of methoxyl group decreased from 17.2 to 8.2, and the percentage of phenolic hydroxyl group increased from 5.2 to 16.0. The decrease of the alcohol hydroxyl group from 13.1 to 10.2 was probably due to the condensation reaction of aliphatic hydroxyl groups as reported previously [32].

The total hydroxyl content was determined by a titration method. The percentage of phenolic hydroxyl or alcohol hydroxyl groups in the total hydroxyl groups can be calculated according to the integral peak areas from NMR spectra using Equations (2) and (3). The phenolic hydroxyl and alcohol hydroxyl content was respectively calculated [39,40] and given in Table 7.

(2) $$R-\text{OH}\left(\%\right)=\frac{R-{\text{OH}}_{\left(\text{H}\%\right)}}{\text{Ar}-{\text{OH}}_{\left(\text{H}\%\right)}+R-{\text{OH}}_{\left(\text{H}\%\right)}}\;\times 100$$

(3) $$\text{Ar}-\text{OH}\left(\%\right)=\frac{\text{Ar}-{\text{OH}}_{\left(\text{H}\%\right)}}{\text{Ar}-{\text{OH}}_{\left(\text{H}\%\right)}+R-{\text{OH}}_{\left(\text{H}\%\right)}}\;\times 100$$

**Figure 9:**
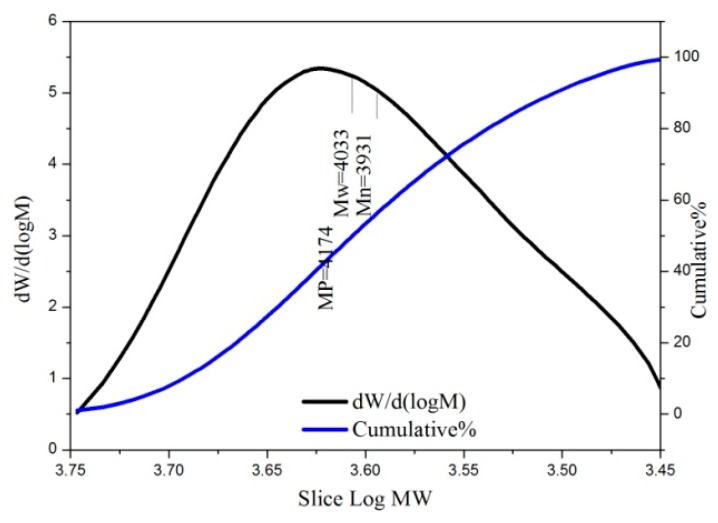
GPC spectrum of D-WSAL prepared under the optimum demethylation condition: 12:1 molar ratio of ICH to lignin, 145 °C, 4 mL DMF, for 3 h under nitrogen atmosphere.

Molecular weight and polydispersity of D-WSAL were analyzed using GPC (Table 8, Figure 9). The weighted average molecular weight of the original WSAL was 6000 g/mol with a polydispersity of 5.3 (provided by the supplier). The results showed that the average molecular weight ($\bar{M_{W}}$) decreased from 6000 to 4033 units and the polydispersity index reduced from 5.1 to 1.0. It indicated that the degradation reaction occurred during the process of lignin demethylation [41], which is in agreement with the results obtained from FTIR semi-quantitative analysis discussed above.

### 3.3. Properties of LPF and D-LPF Adhesives

D-WSAL made by demethylation of WSAL using the optimized process was applied to prepare PF adhesives (D-LPF). In D-LPF synthesis, 60 wt % of phenol was replaced by D-WSAL. The properties of D-LPF adhesives were investigated and compared with adhesives made from WSAL (LPF) as well as PF adhesive without lignin (Table 9). Compared with LPF adhesives, the free formaldehyde content of D-LPF adhesives decreased from 0.65% to 0.22%, the free phenol content decreased from 2.77% to 0.92% and the dry bonding strength increased from 1.13 to 2.28 MPa. The wet bonding strength of D-LPF increased from 0.97 MPa for LPF to 2.11 MPa. Furthermore, the gel time of PF, LPF, and D-LPF was 531, 356, and 243 s, respectively. This may be ascribed to a higher reactivity and a lower molecular weight of the demethylated lignin. However, the gel time can also be the result of other factors such as solids content [42]. The pH values of all adhesives were similar, while the solids content of LPF and D-LPF adhesives were higher than the control PF. However, both the wet bonding strength and dry bonding strength of LPF and D-LPF adhesives were lower than those of the control PF adhesive, and the free formaldehyde content of LPF and D-LPF adhesives increased compared to the control PF adhesive. This may be due to the higher molecular weight and lower reactivity of lignin than phenol [43]. More systematic studies are needed to identify optimum adhesive formulations and processing conditions as well as the impact of the amount of D-WSAL to improve the final performance of D-LPF adhesives.

## 4. Conclusions

Demethylation of WSAL *in-situ* by using a Lewis acid to improve the reactivity of WSAL with formaldehyde in adhesive preparation was investigated in this study. The optimum reaction conditions for the demethylation reaction based on the systematic study of the reaction parameters were as follows: reaction temperature of 145 °C, reaction time of 3 h, molar ratio of ICH to lignin of 12:1, and solvent amount of 4 mL. Moreover, the demethylated lignin (D-WSAL) was used to substitute 60 wt % of phenol in the preparation of lignin containing phenol formaldehyde adhesives (D-LPF). The D-LPF adhesives had a shorter gel time due to the demethylated liginin. Also, the D-LPF contained lower free formaldehyde and free phenol contents, and higher bonding strengths than LPF adhesives with WSAL. Compared with the existing method of demethylation, the reaction condition herein was milder and more economical and environmentally friendly. Therefore, the strategy of using *in-situ* Lewis acid offers a new promising alternative approach for preparing demethylated lignin with a good potential for application in the phenolic formaldehyde adhesives field.

## Figures and Tables

**Table 1:** Results of FTIR semi-quantitative analysis of the relative values of some functional groups of WSAL and D-WSAL calculated with the phenyl group’s skeleton vibration peak at 1503 cm^−1^ as the reference.

ICH/lignin (mol/mol)		Relative values *vs.* reference peak		
		C–H stretching vibration of –CH_3_ and –CH_2_	C–O deformation vibration of CH_3_–O–	C–O stretching vibration of Ar–OH
0:1	peak area ratio	A2,926/A1,506	A1,457/A1,506	A1,200/A1,506
	relative value	1.02	0.82	1.01
5:1	peak area ratio	A2,927/A1,509	A1,462/A1,509	A1,202/A1,509
	relative value	1.34	0.45	2.92
10:1	peak area ratio	A2,926/A1,506	A1,459/A1,506	A1,204/A1,506
	relative value	2.62	0.25	7.21
12:1	peak area ratio	A2,930/A1,505	A1,460/A1,505	A1,205/A1,505
	relative value	3.41	0.17	11.90
15:1	peak area ratio	A2,926/A1,506	A1,459/A1,506	A1,200/A1,508
	relative value	5.70	0.19	10.31

**Table 2:** Relative values of functional groups on D-WSAL prepared with 2.5 mL, 4 mL, and 5.5 mL DMF with 12:1 molar ratio of ICH to lignin, at 145 °C for 3 h under nitrogen atmosphere. (Phenyl group’s skeleton vibration peak at 1503 cm^−1^ was selected as the reference, the relative values of other groups by area ratios of the peaks were calculated.)

DMF/lignin (*w*/*w*)		Relative values based on reference peak		
		C–H stretching vibration of –CH_3_ and –CH_2_	C–O deformation vibration of CH_3_–O–	C–O stretching vibration of Ar–OH
11.9:1	peak area ratio	A2,925/A1,504	A1,452/A1,504	A1,200/A1,504
	relative value	2.68	0.27	7.52
19.0:1	peak area ratio	A2,927/A1,503	A1,454/A1,503	A1,204/A1,503
	relative value	3.41	0.17	11.90
26.1:1	peak area ratio	A2,927/A1,505	A1,457/A1,505	A1,204/A1,505
	relative value	4.02	0.24	9.83

**Table 3:** Relative values of functional groups on D-WSALs synthesized at 135, 145, and 155 °C (phenyl group’s skeleton vibration peak at 1503 cm^−1^ was selected as the reference, the relative values of other groups by area ratios of the peaks were calculated.)

Temperature (°C)		Relative values based on reference peak		
		C–H stretching vibration of –CH_3_ and –CH_2_	C–O deformation vibration of CH_3_–O–	C–O stretching vibration of Ar–OH
130	peak ratio	A2,926/A1,506	A1,456/A1,506	A1,203/A1,506
	relative value	2.98	0.23	7.92
145	peak ratio	A2,927/A1,503	A1,454/A1,503	A1,204/A1,503
	relative value	3.41	0.17	11.90
155	peak ratio	A2,928/A1,503	A1,465/A1,503	A1,206/A1,503
	relative value	4.11	0.22	7.63

**Table 4:** Relative values of functional groups on D-WSALs under nitrogen atmosphere for 7, 5, 3, and 2 h (phenyl group’s skeleton vibration peak at 1503 cm^−1^ was selected as the reference, the relative values of other groups by area ratios of the peaks were calculated).

Time (h)		Relative values based on reference peak		
		C–H stretching vibration of –CH_3_ and –CH_2_	C–O deformation vibration of CH_3_–O–	C–O stretching vibration of Ar–OH
2	peak ratio	A2,927/A1,502	A1,458/A1,502	A1,204/A1,502
	relative value	2.15	0.18	11.00
3	peak ratio	A2,927/A1,503	A1,454/A1,503	A1,204/A1,503
	relative value	3.41	0.17	11.90
5	peak ratio	A2,936/A1,506	A1,456/A1,506	A1,204/A1,506
	relative value	4.06	0.17	11.77
7	peak ratio	A2,926/A1,506	A1,456/A1,506	A1,203/A1,506
	relative value	5.70	0.12	10.31

**Table 5:** Assignments of characteristic absorption peaks from the FTIR spectra of WSAL and D-WSAL.

WSAL band position (cm^−1^)	D-WSAL band position (cm^−1^)	Assignments
3,430	3,438	O–H stretching
2,930	2,927	C–H stretching of methyl and methylene group
2,848	2848	C–H stretching of methoxy group
1,505	1,503	Aromatic skeletal vibration
1,468	1,459	C–O deformation of methoxyl group
1,372	1,373	C–O of syringyl (S) ring
1,264	1265	C–O of guaiacy (G) ring
1,205	1,203	C–O stretching of phenolic hydroxyl group
843	837	C–O of p-hydroxyphenyl (H) propane

**Table 6:** Relative contents of the protons calculated from ^1^H NMR spectra.

Signal (ppm)	H%		Assignment
	WSAL	D-WSAL	
1.90–1.21	9.5	21.7	C–H
2.17–1.96	13.1	10.9	R–OH
2.40–2.17	5.2	16.0	Ar–OH
4.00–3.50	17.2	8.2	CH_3_–O–

**Table 7:** Phenolic hydroxyl and alcohol hydroxyl contents of WSAL or D-WSAL.

Sample	Phenol hydroxyl content (%)	Alcohol hydroxyl content (%)	Total hydroxyl content (%)
WSAL	1.89	4.74	6.63
D-WSAL	6.10	4.16	10.26

**Table 8:** GPC results of D-WSAL prepared under the optimum demethylation condition (the demethylation process was carried out with 12:1 molar ratio of ICH to lignin, prepared at 145 °C with 4 mL DMF for 3 h under nitrogen atmosphere).

Sample	$\bar{M_{n}}$	$\bar{M_{W}}$	Polydispersity
D-WSAL	3,931	4,033	1.03

**Table 9:** Properties of PF, D-LPF, and LPF adhesives.

Sample	pH	Solids content (%)	Free formaldehyde content (%)	Free phenol content (%)	Gel time at 150 °C (s)	Dry bonding strength (MPa)	Wet bonding strength (MPa)
PF	10.2	40.9	0.06%	3.01	531	2.54	2.34
LPF ^a^	10.4	49.2	0.65%	2.77	356	1.13	0.97
D-LPF ^b^	10.4	48.7	0.22%	0.92	243	2.28	2.11

^a^ LPF was prepared by WSAL with a phenol hydroxyl content of 1.89%; ^b^ D-LPF was prepared by D-WSAL with a phenol hydroxyl content of 6.10%.
